# Combining Gamma With Alpha and Beta Power Modulation for Enhanced Cortical Mapping in Patients With Focal Epilepsy

**DOI:** 10.3389/fnhum.2020.555054

**Published:** 2020-12-21

**Authors:** Mario E. Archila-Meléndez, Giancarlo Valente, Erik D. Gommer, João M. Correia, Sanne ten Oever, Judith C. Peters, Joel Reithler, Marc P. H. Hendriks, William Cornejo Ochoa, Olaf E. M. G. Schijns, Jim T. A. Dings, Danny M. W. Hilkman, Rob P. W. Rouhl, Bernadette M. Jansma, Vivianne H. J. M. van Kranen-Mastenbroek, Mark J. Roberts

**Affiliations:** ^1^Department of Cognitive Neuroscience, Faculty of Psychology and Neuroscience, Maastricht University, Maastricht, Netherlands; ^2^Center for Integrative Neuroscience (CIN), Maastricht University, Maastricht, Netherlands; ^3^Neuroscientific MR-Physics Research Group, Department of Diagnostic and Interventional Neuroradiology, Klinikum rechts der Isar, School of Medicine, Technische Universität München, Munich, Germany; ^4^Technical University of Munich Neuroimaging Center (TUM-NIC), Klinikum rechts der Isar, School of Medicine, Technische Universität München, Munich, Germany; ^5^Maastricht Brain Imaging Center (M-BIC), Maastricht University, Maastricht, Netherlands; ^6^Department of Clinical Neurophysiology, Maastricht University Medical Center, Maastricht University, Maastricht, Netherlands; ^7^Basque Center on Cognition, Brain and Language (BCBL), Donostia-San Sebastian, Spain; ^8^Centre for Biomedical Research (CBMR)/Department of Psychology, Universidade do Algarve, Faro, Portugal; ^9^Department of Vision & Cognition, Netherlands Institute for Neuroscience, An Institute of the Royal Netherlands Academy of Arts and Sciences (KNAW), Amsterdam, Netherlands; ^10^Academic Center for Epileptology, Kempenhaeghe/Maastricht University Medical Center, Maastricht, Netherlands; ^11^Department of Neurosurgery, Maastricht University Medical Center Maastricht, Maastricht University, Maastricht, Netherlands; ^12^Donders Institute for Brain, Cognition and Behaviour, Radboud University, Nijmegen, Netherlands; ^13^Grupo Pediaciencias, Facultad de Medicina, Universidad de Antioquia, Medellín, Antioquia, Colombia; ^14^School for Mental Health and Neuroscience (MHeNS), Maastricht University, Maastricht, Netherlands; ^15^Department of Neurology, Maastricht University Medical Center, Maastricht, Netherlands

**Keywords:** electrical stimulation mapping, broadband gamma frequency, alpha frequency band, beta frequency band, drug-resistant epilepsy, epilepsy surgery, electrocorticography-based functional mapping, frequency-based cortical mapping

## Abstract

About one third of patients with epilepsy have seizures refractory to the medical treatment. Electrical stimulation mapping (ESM) is the gold standard for the identification of “eloquent” areas prior to resection of epileptogenic tissue. However, it is time-consuming and may cause undesired side effects. Broadband gamma activity (55–200 Hz) recorded with extraoperative electrocorticography (ECoG) during cognitive tasks may be an alternative to ESM but until now has not proven of definitive clinical value. Considering their role in cognition, the alpha (8–12 Hz) and beta (15–25 Hz) bands could further improve the identification of eloquent cortex. We compared gamma, alpha and beta activity, and their combinations for the identification of eloquent cortical areas defined by ESM. Ten patients with intractable focal epilepsy (age: 35.9 ± 9.1 years, range: 22–48, 8 females, 9 right handed) participated in a delayed-match-to-sample task, where syllable sounds were compared to visually presented letters. We used a generalized linear model (GLM) approach to find the optimal weighting of each band for predicting ESM-defined categories and estimated the diagnostic ability by calculating the area under the receiver operating characteristic (ROC) curve. Gamma activity increased more in eloquent than in non-eloquent areas, whereas alpha and beta power decreased more in eloquent areas. Diagnostic ability of each band was close to 0.7 for all bands but depended on multiple factors including the time period of the cognitive task, the location of the electrodes and the patient’s degree of attention to the stimulus. We show that diagnostic ability can be increased by 3–5% by combining gamma and alpha and by 7.5–11% when gamma and beta were combined. We then show how ECoG power modulation from cognitive testing can be used to map the probability of eloquence in individual patients and how this probability map can be used in clinical settings to optimize ESM planning. We conclude that the combination of gamma and beta power modulation during cognitive testing can contribute to the identification of eloquent areas prior to ESM in patients with refractory focal epilepsy.

## Highlights

- Gamma, alpha and beta band activity have significant diagnostic ability to identify electrical stimulation mapping (ESM)-defined eloquent cortical areas.
- We present a novel method to combine gamma and lower frequency activity for enhanced identification.
- We quantify how identification is dependent on analysis time window, cortical function, and patient’s attentional engagement.
- We show how ECoG responses can be used to make a probabilistic map, useful for the improvement of patient-specific planning of ESM.

## Introduction

Invasive cortical mapping for the precise characterization of “eloquent” cortical areas is necessary to minimize neurological or cognitive complications following resection of pathological tissue. In the current gold standard “electrical cortical stimulation mapping” (ESM, Penfield and Boldrey, 1937; Hamberger, 2007), electrical stimulation of subdural electrode-pairs that disrupts function (e.g., speech production) or produces neurological symptoms (e.g., paraesthesia) indicates that the stimulated cortex is “eloquent” and should be preserved during resection. Despite its usefulness (Ojemann et al., 1989), ESM can elicit after-discharges, seizures (Lesser et al., 1984; Lee et al., 2010), or pain (Lesser et al., 1985). Furthermore, it requires the patient’s continuous compliance, rendering it challenging to use in some patients (Arya et al., 2015). ESM is also time consuming, requiring individual testing of each implanted electrode-contact, restricting the number of electrodes that can be tested and precluding the use of high density arrays (Bouchard et al., 2013; Mesgarani et al., 2014; Muller et al., 2016) thereby limiting the spatial resolution of ESM (Hermiz et al., 2018). These and other factors motivate the search for alternative ways to identify eloquent cortex (Crone et al., 2006, 1998a,b; Brunner et al., 2009; Lachaux et al., 2007; Vansteensel et al., 2010).

Activation of neuronal networks leads to a change in the spectral power of electrical field potentials of local neuronal populations (Buzsaki, 2004; Fries et al., 2007; Buzsáki et al., 2012). For example, an enhancement of gamma band power (>30 Hz) during visual (Gray et al., 1989) and auditory (Brosch et al., 2002) processing, motor preparation (Pfurtscheller et al., 1993; Vansteensel et al., 2013), and sensorimotor integration (Murthy and Fetz, 1992). Gamma power varies with high temporal and spatial resolution, such that increasing gamma power is specific to active neuronal populations (Crone et al., 1998a, 2006; Aoki et al., 1999, p. 199; Sinai et al., 2005; Leuthardt et al., 2007; Miller et al., 2007; Nagasawa et al., 2010; Wu et al., 2010; Buzsáki et al., 2012; Arya et al., 2018; Hamilton et al., 2018). Consequently, gamma modulation has been proposed as an alternative for ESM (Crone et al., 1998a, 2006; Aoki et al., 1999; Lachaux et al., 2003; Sinai et al., 2005; Leuthardt et al., 2007; Miller et al., 2007; Brunner et al., 2009; Wu et al., 2010; Vansteensel et al., 2013; Wang et al., 2016; Arya et al., 2017) whereby a task-dependent increase in gamma power indicates eloquent cortex. However, results are mixed and gamma band-based mapping typically has insufficient accuracy in replicating ESM results, with some studies (Wu et al., 2010) reporting high sensitivity but low specificity, while others report the opposite (Bauer et al., 2013). In a recent review Arya et al. concluded that mapping based on gamma was not sufficient, and also highlighted the heterogeneity in the diagnostic threshold and the cognitive task employed (Arya et al., 2018). Thus, there is scope for improvement both in the implementation and interpretation of ECoG response based mapping.

Activation of neuronal networks is typically accompanied by a reduction in power in the alpha (8–12 Hz) and beta (15–25 Hz) frequency bands. Recent empirical evidence demonstrates that alpha and beta power is related to active inhibition processes (Jensen and Mazaheri, 2010) and can be highly spatially specific to activated populations (de Pesters et al., 2016; Muller et al., 2016). Thus, power modulation in lower frequency bands represents a plausible source of additional information for cortical mapping. Moreover, given that different frequency bands have different functions (Scheeringa and Fries, 2017) and are not directly correlated to each other (Bonaiuto et al., 2018), additional information may come from the combination of bands. A limited number of studies have investigated the use of lower frequency bands for cortical mapping (Crone et al., 1998b; Sinai et al., 2005; Leuthardt et al., 2007; Wu et al., 2010; Hermes et al., 2012; Bauer et al., 2013; Vansteensel et al., 2013). The results from these studies have been variable in terms of the reported diagnostic ability of the low frequency bands. Some studies have also investigated the combination of frequency bands (Leuthardt et al., 2007; Wu et al., 2010). These studies showed that accepting electrodes with a high response in either the gamma band, the beta band, or in both, as potentially eloquent yielded better results than using either band alone, especially in the language cortex. Likewise, machine- and deep- learning approaches (Prakash et al., 2017; RaviPrakash et al., 2020) have shown that using the entire frequency spectrum, rather than only the gamma band, yields better mapping results, although it was not made clear which part of the spectrum offered the most additional information. In addition, while there is considerable heterogeneity in the task employed between studies, few studies have directly compared different tasks in the same group of patients. Thus, little is known about how the cognitive engagement of the patient, i.e., the task performed during cortical mapping, impacts the quality of ECoG based mapping.

We hypothesized that alpha and beta band power modulation, either on their own or in combination with the gamma power modulation, could enhance the accuracy of the identification of eloquent cortex compared with the use of gamma alone. To investigate this, we registered (recorded) ECoG signals from subdural electrodes in 10 patients with drug-resistant focal epilepsy who underwent ESM. Patients performed a delayed match-to-sample (DMTS) task or listened to the same stimuli without an active task. We show that beta power generally had equal diagnostic ability to gamma, while alpha power was less effective. Withdrawing attention from the stimuli reduced the diagnostic ability. The combination of gamma and beta frequency bands, using a Generalized Linear Model (GLM) was consistently better than either band individually.

## Materials and Methods

### Participants

We included 10 patients (age: 35.9 ± 9.1 years, range: 22–48, 8 females, 9 right handed, native Dutch speakers with normal cognitive ability, with normal or corrected to normal vision, and normal hearing) with drug-resistant focal epilepsy, who underwent continuous, extraoperative ECoG with subdural electrodes and ESM as part of pre-surgical evaluation for resective epilepsy surgery. The electrode implantation scheme was strictly chosen according to the clinical criteria (see Supplementary Material section “1.1 Electrode implantation procedure”) for a complete implantation description of all patients. All patients scheduled for epilepsy surgery were invited to join the study during preoperative consultation. No financial incentive for joining the study was given. Those who volunteered as participants for the study signed an informed consent form and performed the DMTS task similar to that used in Archila-Meléndez et al. (2018) (see Supplementary Material section “1.4 Delayed Match-to-Sample Task”). The used audiovisual stimuli and the delayed match-to-sample (DMTS) task performed by the patients were designed to test hypotheses about the processing of acoustic properties of speech in the language network under conditions of varying selective attention, yet we reasoned that, considering the multimodal nature of the task which engaged language listening, reading, working memory and a motor response, the ECoG data collected during the performance of the DMST task might be also useful to test our current hypothesis. The task was performed during the first 5 days after surgical implantation, when clinical condition (alertness, pain level, no recent seizure activity) allowed and when the patient was willing to participate. One patient performed poorly in the DMTS task (Table 1), however her data was included in our analyses. The study complies with the Declaration of Helsinki for research studies in humans and was approved by the Medical Ethical Committee of the Maastricht University Medical Center, Maastricht, The Netherlands.

**TABLE 1:** Demographic information and clinical summary of the patients included in the study.

Patient	Age (y)	Sex	Electrode implantation scheme	Seizure frequency	Cognitive status	Education	Wada	fMRI	Handedness	% correct trials	Total number of trials
1	42	F	Grid temporal L	Daily	Average*	Medium professional	Not performed	Not performed	Right	90	1,126
2	39	M	Grid temporal L	Weekly	Low average*	Lower vocational	Not performed	Left dominance	Right	87.1	644
3	22	F	Grid temporal L	Daily	High average	Higher professional	Not performed	Right dominance	Right	98.5	810
4	28	F	Grid frontal L	Twice per week	Average	Higher professional	Not performed	Left dominance	Right	89.3	486
5	33	F	Strips ventro- latero-temporal L and R	Daily	Average*	Lower vocational	Not performed	Not performed	Right	93.3	972
6	38	M	Grid temporal R	Weekly to daily	Low averge	Higher professional	Right dominance	Not performed	Left	96.6	1,134
7	23	F	Grid temporal and perieto-frontal L	Weekly	Average	Higher professional	Not performed	Left dominance	Right	99	810
8	39	F	Grid parieto-Occipital R	Daily	No data	Medium professional	Not performed	Not performed	Right	96.9	773
9	48	F	Grid temporal and perieto-frontal L	Twice per month	Average	Lower vocational	Not performed	Not performed	Right	53.8	236
10	47	F	Grid parieto- temporo-occipital + ventro-temporal strips R	Twice per month	Average	Medium professional	Not performed	Not performed	Right	98.3	810

*Deterioration from previous evaluation. L: left, R: right.

### Electrical-Cortical Stimulation Mapping (ESM)

ESM was performed using bipolar stimulation between electrode pairs sequentially, selecting neighboring electrode pairs of the subdural grid and/or strips. The procedure for ESM differs between centers, with consequences for the reproducibility of comparisons between ESM and ECoG response-based mapping approaches (Mooij et al., 2018). Therefore, we provide a detailed description of our ESM procedure in Supplementary Material. No patients included in the study experienced immediate evident impairments following resection surgery.

### Data Pre-processing, Time-Frequency Analysis and Filtering

Data were analyzed in MATLAB (R2017b version 9.3.0.713579; The Mathworks Inc.; Natick, MA, United States) using the FieldTrip toolbox (Oostenveld et al., 2011) and custom scripts. Data were first cut into epochs from 1 s before the sound onset until 1 s after the behavioral response with a maximum data length of 8 s. We then applied a discrete time filter at 50, 100, and 150 Hz to remove line noise and down-sampled the data from 2048 to 500 Hz. Data were re-referenced to the average signal recorded in all electrodes, after excluding electrodes with high noise.

Time-frequency representations (TFR) were calculated in 2 Hz steps from 6 to 250 Hz using Hanning tapers (7 cycles) and 10 ms step size, 6 Hz being the lowest frequency that could be reliably measured in the trial structure composing our DMTS task. Power was expressed as the normalized change, where the change was defined as the difference between post-stimulus time window and pre-stimulus “baseline” period -700 to -100 ms before sound onset. To better represent the spectral response around each trial event, TFRs were aligned and cut around the onset of each event. Thus, we represent −0.5 to 1 s around the onset of the sound, −0.2 to 0.7 s around onset of the letters and −0.3 to 0.7 s around the onset of the behavioral response. For frequency specific analysis, data were filtered at alpha (8–12 Hz), beta (15–25 Hz) and broad gamma band (55–200 Hz) using a 4th order Infinite impulse response (IIR) Butterworth two-pass filter. Power was calculated per trial as the absolute values of the Hilbert transform and the resulting time courses were expressed as normalized power change. For non-time resolved analysis, average power was first calculated per trial and then across trials.

### Generalized Linear Model (GLM) Fitting and Validation

We aim at testing if a linear combination of different frequency bands results in a better diagnostic ability, as compared to single frequency bands. We used a GLM with binomial likelihood and logit link function (McCullagh and Nelder, 1998), where for each electrode the dependent variable (y) was the ESM category (i.e., eloquent and non-eloquent) and the predictors were the ECoG power change in each frequency band. The estimated model was used to determine the optimal weighting of power change per frequency band as regression coefficients to predict the ESM response. The process was performed in k-fold cross-validation, in k-1 partitions. The resulting model was used to predict the ESM category in a remaining k-partition. The process was then repeated 20 times with random partitions and compared with the ground truth ESM and the results were averaged (see details in Supplementary Material).

### Area Under the Receiver Operating Characteristic Curve Analysis and Bootstrap Procedure for Statistical Testing

We aimed to calculate the diagnostic ability of the three frequency bands, and of their combination. We calculated the area under the curve (AU) from the receiver operating characteristic (ROC) curve (Green and Swets, 2000). This procedure calculates the ratio of sensitivity to specificity for all potential discrimination thresholds. The resulting AUROC represents the maximum performance (i.e., correct discrimination) of an “ideal observer” using the optimal threshold. To facilitate the comparison of AUROC with changes in power spectrum for alpha and beta frequencies, we display significant AUROC values below 0.5 where power in eloquent channels is suppressed more strongly than power in non-eloquent channels (see Supplementary Material section “1.6 Area Under the Receiver Operating Characteristic Curve Analysis”). Foe statistical testing we implemented a bootstrap procedure with replacement for statistical testing in which we constructed a distribution of AUROC values for each model (i.e., frequency bands and their combination; see Supplementary Material section “1.7 Bootstrap Procedure for Statistical Testing Receiver Operating Characteristic Between Models”).

### Function-Specific and Attention-Dependent Analyses

We were interested in studying whether different functional areas might exhibit characteristic power spectra, however we were limited by the low numbers of electrodes representing some ESM categories. We therefore grouped electrodes into two broad categories, which we call “input,” being mostly involved in processing cortical inputs (i.e., sensory processing) and “*output*” as being mostly concerned with cortical *outputs* (motor function). This categorization is based on the concept that one of the nervous system’s fundamental functions is the processing of sensory input to guide behavioral (motor) *output*. Those electrodes labeled as *auditory, visual, sensory, language-Wernicke*, and *language-temporobasal* were grouped into the “*input*” category as being more functionally involved in processing incoming sensory events. Those labeled as *motor*, *mixed-sensorimotor*, and *language-Broca* were grouped into the “*output*” category as being more involved in generating behavioral responses. Two neighboring electrodes in one patient labeled as *emotion* were excluded from the function-specific analysis as they did not seem to fit in either category.

### Anatomical Segmentation and Intracranial Electrode Localization

**FIGURE 1:**
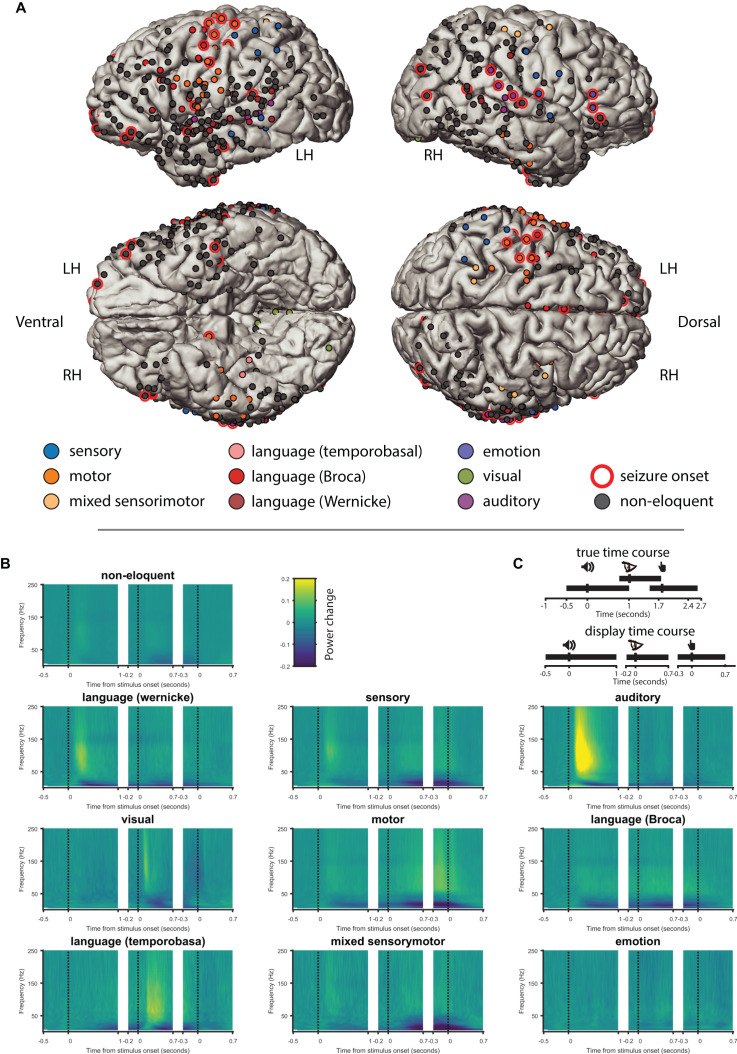
ESM categories and ECoG responses. **(A)** Electrodes tested during electrical-cortical stimulation mapping (ESM) in 10 patients projected in the common space, color coded according to the ESM category. LH, left hemisphere; RH, right hemisphere; Ventral, ventral (caudal) view of left and right hemispheres; Dorsal, dorsal (cranial) view of left and right hemispheres. **(B)** Time frequency representations (TFR) of time periods of the delayed match-to-sample task from all tested electrodes grouped by the ESM category. The TFRs represent the three different trial events (i.e., sound onset, letter onset, and button-press), which are illustrated by the three vertical dotted lines in each TFR). **(C)** Illustration of the time course in the task (upper cartoon, events are represented at their average time after sound onset) and the “exaggerated” time between the events used for display time (lower cartoon).

Presurgical T1-weighted MRI were segmented using FreeSurfer (version 6.0)^1^ (Dale et al., 1999; Fischl et al., 1999a). Briefly, this procedure includes removal of non-brain tissue using a hybrid watershed/surface deformation method, automated Talairach transformation, intensity normalization, tessellation of the gray/white matter boundary, automated topology correction, and surface deformation following intensity gradients. After the process was finished, quality control was performed by visually inspecting each subject’s brain and the overlay of the tissue boundaries. Remaining errors were manually corrected using ITK-SNAP software (version 3.4.0^2^; Yushkevich et al., 2006). After segmentation, the postsurgical CT scan was coregistered to the presurgical T1-weighted MRI using rigid affine transformations via FSL’s FLIRT algorithm (Jenkinson and Smith, 2001). To localize the intracranial electrodes, we used the MATLAB toolbox iELVis following the procedure described by Groppe et al. (2017). Briefly, the locations of the electrodes in the postsurgical CT scan were manually identified using intensity thresholding in BioimageSuite^3^. To correct for post-implantation brain shift, electrodes were projected to the nearest point on the dural surface reconstructed from the presurgical MRI (Dykstra et al., 2012). Finally, for visualization purposes, electrodes of all patients were projected to the brain average surface of FreeSurfer (Fischl et al., 1999b, see Figure 1A using iELVis; Groppe et al., 2017).

### Probabilistic Map of Eloquence

We considered how our analysis could be clinically useful without providing a diagnostic test. A probabilistic map, that shows the likelihood of eloquence for each electrode and that does not require the ESM labels but only the ECoG signal during the DMST task (and thus could be obtained before the ESM), could be used to optimize the planning of the sequence of electrode pairs for ESM testing. We took GLM-calculated probabilities for each patient separately using only data from the remaining patients to calculate the GLM beta weights. This procedure represents a special case of our standard analysis in which data was separated into training and test datasets: In the standard analysis the separation was done randomly, thus data from all patients could be included for training and the same could be done for testing; in the current analysis the data separation was performed according to the patient identity. In this way we show how a probabilistic map of eloquence during ESM can be generated for an individual patient without reference to the ESM results of that patient. This procedure allows, in a new patient, the creation of a probabilistic map of eloquence using only data from the DMTS task that is acquired before the ESM, enabling the incorporation of the probabilistic map in the planning of the ESM.

## Results

### Relationship Between Electrical Cortical Stimulation and Frequency Modulation

As a descriptive analysis, we selected all electrodes from all ESM categories as defined by the electrophysiologist (see Supplementary Material) and projected them into a common brain space (Figure 1A). We registered in total 129 eloquent and 443 non-eloquent electrodes (Table 2). Eloquent electrodes were located bilaterally over the temporal neocortex (superior and middle temporal gyri), over the inferior frontal gyrus, and over the pre- and post-central gyri.

**TABLE 2:** Electrode details.

Patient	Total stimulated	Total non-eloquent	Total eloquent	Seizure onset	Sensory	Motor	Mixed sensorimotor	Language (Wernicke)	Language (Broca)	Language (temporobasal)	Auditory	Visual	Emotion
1	39	35	4	5	2	0	0	1	1	0	0	0	0
2	39	39	0	2	0	0	0	0	0	0	0	0	0
3	40	29	11	0	4	0	0	1	0	0	6	0	0
4	74	59	15	12	0	8	0	0	7	0	0	0	0
5	29	29	0	0	0	0	0	0	0	0	0	0	0
6	40	26	14	8	4	2	0	1	0	2	3	0	2
7	79	43	36	7	5	14	2	7	7	0	1	0	0
8	74	63	11	3	1	0	3	1	0	0	2	4	0
9	80	57	23	1	2	14	3	3	1	0	0	0	0
10	78	63	15	3	4	8	0	0	0	0	0	3	0
Total	572	443	129	41	22	46	8	14	16	2	12	7	2

*Number of electrodes in each ESM functional category, electrically stimulated and seizure onset per patient.*

We represented the change in power spectral density, relative to the baseline period (−0.7 to −0.1 s from sound onset) across time (time-frequency representation, TFR) of the ECoG signal during each epoch of the DMTS task (Figure 1B). ESM categories exhibited characteristic response patterns during the task. For example, electrodes labeled as “*auditory*” exhibited increased gamma activity and decreased alpha/beta power after sound onset. Likewise, electrodes labeled as “*visual*” exhibited increased gamma and decreased alpha/beta after letter onset. Electrodes labeled as “*motor*” exhibited an increase gamma activity and a decrease in alpha/beta around the button press. Interestingly, “*language temporobasal*” and “*language Broca*” electrodes were active after sound onset and letter presentation possibly pointing toward covert rehearsal of the sound and silent reading of the letters. For further analysis, we grouped all electrodes labeled as eloquent for comparison with all electrodes that were tested using ESM but labeled as non-eloquent.

### Receiver Operating Characteristic (ROC) Curve Analysis

**FIGURE 2:**
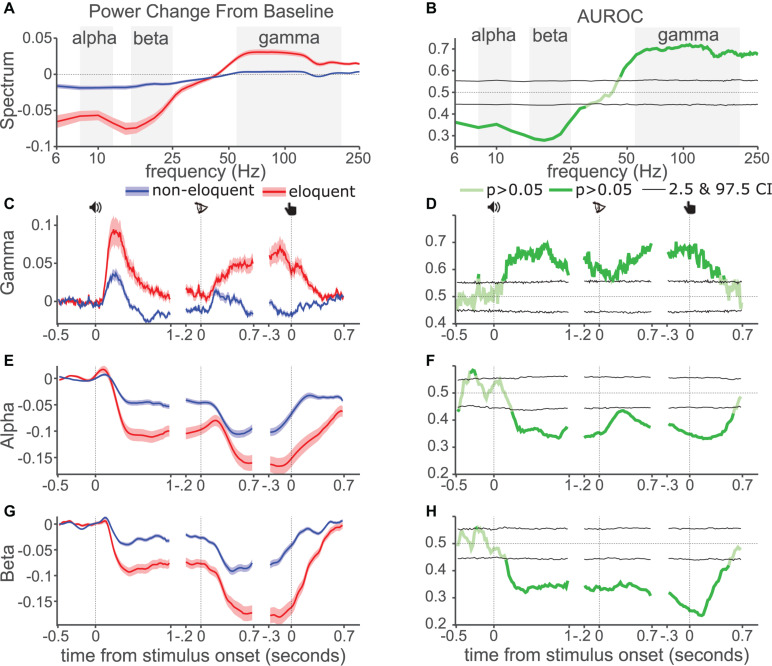
AUROC analysis. **(A)** Normalized change in ECoG signal power spectral density from baseline from eloquent (red) and non-eloquent electrodes (blue). Shading shows standard error, center line shows mean. **(B)** AUROC values across frequencies, pale green, non-significant, dark green significant. Black lines show 2.5 and 97.5 percentiles from permutation distribution. Dotted line shows chance performance. Background shading indicates filter boundaries for each band. **(C)** Gamma power modulation during each task event (icons indicate sound onset, letter onset, and button-press). **(D)** AUROC values across time for gamma. **(E,F)** Alpha power and AUROC values, as in **(C,D)**. **(G,H)** Beta power and AUROC values, as in **(C,D)**.

We compared the power spectrum of eloquent and non-eloquent electrodes’ ECoG during the DMTS task. Power was generally increased relative to baseline for frequencies above 50 Hz, and generally decreased for frequencies below 30 Hz (Figure 2A). This effect was larger in eloquent- (red) than non-eloquent electrodes (blue). To test the diagnostic ability of this difference we calculated AUROC (Figure 2B) and applied a permutation test for statistical assessment. AUROC was significant (darker line, *P* < 0.05, two-sided test, uncorrected for multiple comparisons) for frequencies between 50 and 180 Hz and for frequencies between 6 and 30 Hz, indicating significant diagnostic ability, with a performance of 65–70% for an ideal observer.

To represent the time courses of gamma-, alpha- and beta bands over the trial we filtered the ECoG signal in each band (gray background shading, Figures 2A,B) and calculated the absolute value of the Hilbert transform, which was subsequently represented as the normalized change against the pre-sound baseline. We found stronger gamma increase in eloquent than non-eloquent electrodes (Figure 2C) during the three task epochs. The AUROC values across time (Figure 2D) were larger than 0.5 and significant for all three events.

Alpha power (Figure 2E) decreased in all three events (Figure 2E) especially in eloquent electrodes. The AUROC values were significantly below 0.5 for all three events (Figure 2F) indicating that power for eloquent electrodes was lower than non-eloquent electrodes according to the definition of our AUROC calculation (see Supplementary Material section “1.6 Area Under the Receiver Operating Characteristic Curve Analysis”). A similar pattern was observed for beta power (Figures 2G,H), however, the decrease in power was especially prominent around the button-press. AUROC values were generally lower for beta than for alpha band, indicating greater diagnostic ability for beta, especially around the button-press.

**FIGURE 3:**
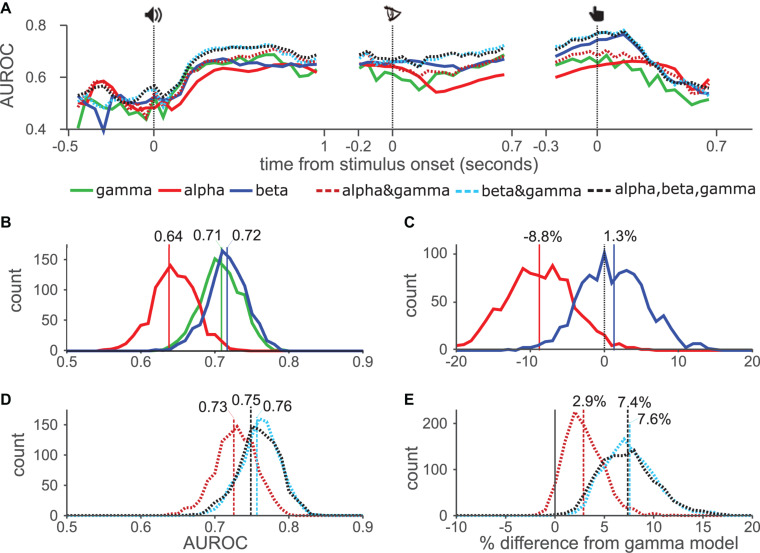
Temporal AUROC and model comparison. **(A)** AUROC values using GLM approach for gamma, alpha, and beta (solid lines), and their combinations (dashed lines). **(B)** Distribution of the AUROC values from 1000 bootstrap samples for single models. Vertical lines show AUROC values without resampling. **(C)** Pairwise comparison from bootstrap distribution of alpha (red) and beta (blue) AUROC values against gamma. Vertical lines show mean percentage change from gamma-only model. **(D,E)** Distribution of AUROC values for combination models, as in **(B)**. **(E)** Pairwise comparisons of combination models with gamma-only model.

Next, we combined the responses from different bands using a GLM including 10-fold cross-validation (methods) and calculated AUROC values across time using the GLM response (Figure 3A). For smoothing, and to reduce processing time, we used a sliding time window of 100 ms with 50 ms step size. The GLM based AUROC values for gamma-only closely matched standard AUROC values (Figure 3A, green line). For alpha-only (red) and beta-only (blue) the GLM based AUROC values also closely matched the standard AUROC, except that values were above 0.5 rather than below due to the fitting procedure. Interestingly, time courses for the three individual band models peaked at different times in the trial. The gamma-only model performed best after the sound onset while the beta-only model performed best around the button-press. The alpha-only model performed generally worse than the other models, but interestingly outperformed the gamma-only model around the letter onset and late after the button-press. The combined model AUROC values (Figure 3A–dashed lines) tended to be either higher than, or equal to, whichever individual curve was highest at any given time point. This was especially true for the beta&gamma model (blue dashed lines), although the alpha&gamma (red dashed) model also tended to outperform the gamma-only model. Interestingly the three-band model (black dashed) did not outperform the beta&gamma model indicating that including alpha brought no additional information.

To statistically test the AUROC results of each model against chance performance and against each other, we first reduced the dimensionality of the data by calculating average power for each electrode, collapsing over the time dimension. Using this data we conducted a bootstrap analysis in which we constructed a distribution of 1000 AUROC values for each model by creating multiple datasets of the same size as the original, while randomly drawing electrodes with replacement. For each dataset, and the original, we computed the AUROC for each model using 10-fold cross-validation. Histograms in Figure 3B show the distribution of AUROC values of the single band models, vertical lines show the AUROC values from the original dataset. All three models performed better than chance, with 100% of the resampled datasets returning AUROC values greater than 0.5. Of the three models the alpha-only model (red) performed the worst, with an AUROC of 0.64 in the original dataset, the gamma-only (green) and beta-only (blue) performed similarly. The gamma-only approach has previously been used most widely, therefore, to test the usefulness of alpha- and beta bands we made pairwise comparisons of those models against the gamma-only model (Figure 3C), i.e., using the same selection of randomly chosen channels for each model. The alpha-only model was worse than the gamma-only model in 98% of datasets, which we consider to show a significant difference. The mean performance reduction was 8.8% (vertical red line). While the beta-only model offered a slightly increase performance (1.3%), which was not significant. We next compared the performance of the combination models (Figure 3D). These generally outperformed the individual-band models although the alpha&gamma (red dashed) model performed the worst. The beta&gamma (blue dashed) and three-band (black dashed) model performed equally. In pairwise comparisons with the gamma-only model, all three models offered enhanced performance although this just failed to meet the threshold of significance for the alpha&gamma model (94% of datasets improved). The beta&gamma and three-band models both performed significantly better than the gamma-only model (in 100% of datasets) offering respectively 7.6 and 7.4% average improvement. Their distributions overlapped indicating again, that there was no advantage to including the alpha band to the beta&gamma model.

Taken together these results show that the alpha-only model generally performed worse than the other single-band models, whereas the beta-only and gamma-only models performed about equally. Combining alpha with gamma provided some improvement, whereas combining beta and gamma provided a larger improvement.

### AUROC Performance Depends on Functional Category

Alpha-, beta- and gamma bands have been ascribed different functional roles, with gamma associated with feedforward processes and alpha associated with feedback (Scheeringa and Fries, 2017). The beta-band has been associated with motor activity, whereby beta power drops in preparation for motor output. Thus, these bands may have relatively different importance in different cortical areas, depending on the area’s place in the cortical hierarchy (Felleman and Van Essen, 1991). Specifically, we can anticipate that input areas, early in the hierarchy, may have a high dependence on gamma, while output areas, late in the hierarchy may be more dependent on alpha and beta. Importantly, this implies for our current question that, in patients with many output electrodes, alpha or beta may be more informative, or their combination with gamma may give greater improvement.

**FIGURE 4:**
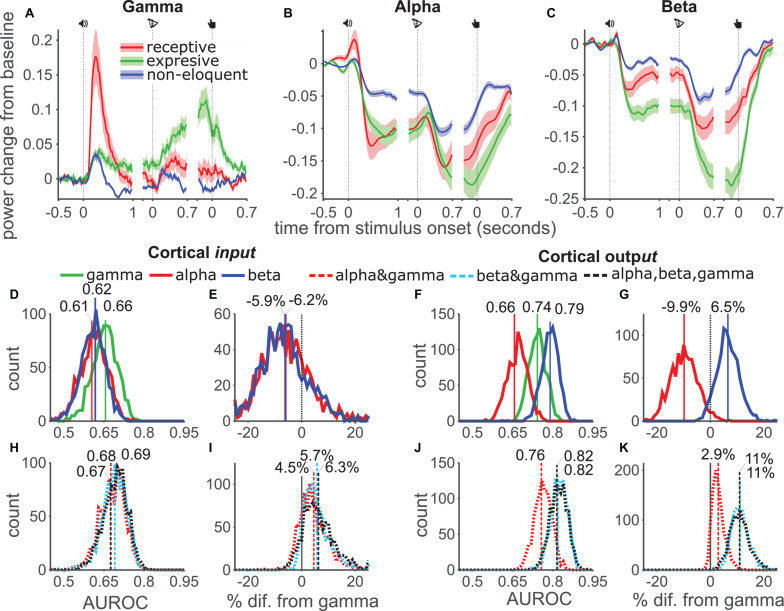
AUROC analysis per functional category. **(A–C)** Gamma, Alpha and Beta power modulation and SE (shades) for three subtypes of electrodes (input, output and non-eloquent) during the three events of the task. **(D)** Distribution of the AUROC values from 1000 bootstrap samples for single models and input channels. **(E)** Pairwise comparison from bootstrap distribution of alpha (red) and beta (blue) AUROC values against gamma for input channels. **(F,G)** Same as **(D,E)** for output channels. **(H–K)**, same as **(D–G)** using combined models.

To test this hypothesis we divided all eloquent electrodes into two broad categories which we defined as output and input and repeated our analysis (Figure 4) for each group. Notice that this grouping was intended as a procedural means to split the data into denominated groups. With the possible exception of primary areas, all cortical areas have roles in both processing incoming stimuli and generating responses, thus the distinction between output and input areas is only an approximation.

Descriptively, gamma power increased in the first task epoch for all electrodes (Figure 4A). Input electrodes (red) showed the highest power followed by output electrodes (green). Non-eloquent electrodes (blue) showed the weakest response. After letter onset, gamma power increased for all electrodes. While power in input electrodes peaked soon after letter onset, power in output electrodes continued to increase, peaking shortly before the button-press. Non-eloquent electrodes showed low power after a weak response to letter onset and button-press. Alpha power (Figure 4B) was suppressed in all channels with greater suppression for eloquent than non-eloquent channels. Interestingly, alpha power suppression in both output and input channels was approximately equal for the majority of the time course. Only at the end of the trial did output channels show somewhat greater suppression. By contrast, beta band (Figure 4C) suppression was considerably stronger in output channels than input channels at all-time points, especially after the button press. With our stated caveat about the distinction between output and input areas, these data supported our grouping, as “*input*” electrodes seemed most involved during stimulus processing and “output” electrodes seemed most involved near the behavioral response.

We calculated AUROC values for the three bands separately and for the three combination models using the time-averaged responses and tested significance of the difference using the bootstrap method. In *input* (Figure 4D) electrodes the distribution of gamma (green) AUROC values was higher than the alpha-only (red) or beta-only (blue) models, however the distributions heavily overlapped and this difference was not significant in pairwise comparisons (Figure 4E). In *output* electrodes (Figure 4F) the beta-only model outperformed both gamma-only and alpha-only models. Pairwise comparisons (Figure 4G) showed that this difference corresponded to a 6.1% increase but did not pass significance (93% of datasets showed an increase). The alpha-only model performed significantly worse than the gamma-only model, corresponding to a 11% drop.

Analysis of the combination models showed that in *input* channels all three models offered approximately equal performance (Figure 4H), which was somewhat, but not significantly, better than the individual band models (Figure 4I). Among *output* channels the combination models offered better performance (Figure 4J) and a greater improvement over the gamma-only model (Figure 4K): The alpha-gamma model mean improvement was 3% but failed to reach significance, while the beta-gamma and three-band models both showed an improvement in 100% of dataset with a mean improvement of 11%. These results were in line with our expectation that *output* electrodes, being higher in the cortical hierarchy, would be most sensitive to low frequency power modulations. Taken together these results suggest that eloquent areas may be best mapped using gamma or beta-band power depending on the distribution of electrodes in an individual patient. However, irrespective of the ESM functional category, the combination of beta and gamma bands was reliably the best measure for the identification of eloquent electrodes.

### Influence of Attention on AUROC Values

**FIGURE 5:**
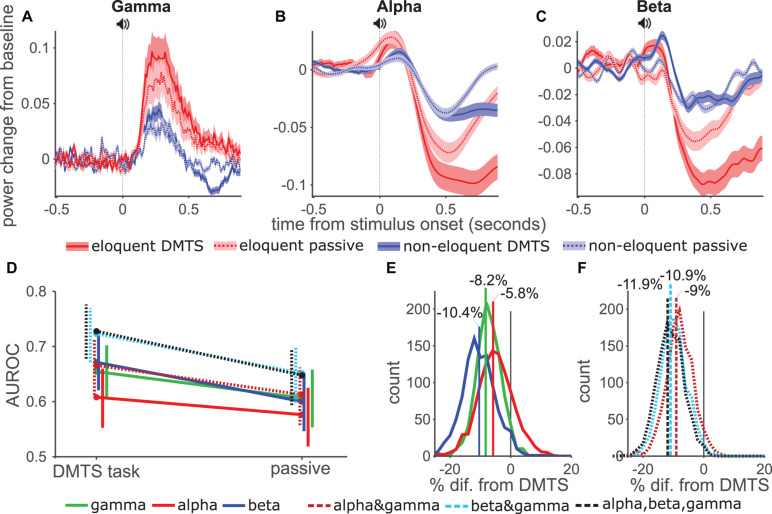
Influence of attention on AUROC values. **(A–C)** Gamma, Alpha and Beta power across time for eloquent (red) and non-eloquent (blue) electrodes during the active (DMTS, solid lines, darker shading-SE) and the passive (dashed lines, lighter shading-SE) tasks. **(D)** Effect of attention on diagnostic ability per model. Vertical lines show 5 and 95 percentiles from 1000 bootstrap samples, horizontal offset to aid visibility. **(E)** Pairwise comparison between active and passive tasks per single band models. Negative values imply a better performance in the active task. **(F)** Same as **(E)** but using combination models.

Most patients performed well in the task, however, one patient performed poorly and withdrew early from the experiment. This experience prompted us to question how well ECoG mapping would perform in a less demanding task, which might be particularly relevant when applying ECoG mapping in pediatric populations, patients with low general cognitive ability (Ruiz-Rizzo et al., 2018, 2019) or patients who are otherwise unable or unwilling to engage in demanding cognitive testing (Haupt et al., 2020; Ruiz-Rizzo et al., 2020). Our cognitive experiment included a passive condition in which the same auditory stimuli were presented without any explicit task. We repeated our analysis using the ECoG response, from eloquent (red, Figure 5) and non-eloquent electrodes (blue), during the sound presentation in the active (solid lines) and passive tasks (dashed lines, Figure 5). Gamma band (Figure 5A) responses were weaker in the passive task compared to active task for both eloquent and non-eloquent electrodes. Alpha and beta band suppression (Figures 5B,C) was weaker in the passive task compared to the active task, especially late in the response.

As before, we calculated AUROC values of the GLM models and tested significance using the time-averaged responses, however, here we used only the period from sound onset to 900 ms which could be compared for both tasks. In the active task AUROC values were unsurprisingly lower than in previous analysis (Figure 4H) where we had used the response during the entire trial. However, when comparing the performance of the three bands and the combination, the pattern matched our previous findings. Comparing the two tasks (Figure 5D) showed that withdrawing attention in the passive task lowered performance of all models. In pairwise comparisons between the two tasks we found that performance of the models dropped by as much as 11.9% in the three-band models (Figure 5F). The drop was significant (*p* < 0.05) for all models except the alpha-only model (*p* = 0.19).

These data showed that an active task which engages attention is required for optimal mapping using ECoG, with important implications for studies which attempt to classify eloquent cortex without explicit cognitive tasks (Vansteensel et al., 2013). Nevertheless, the GLM approach combining gamma and beta band activity offered a significant improvement over single-band models.

### Probabilistic Map of Likely Eloquence

We aimed to use the GLM fitting to calculate a map showing the probability that each electrode would be eloquent given the ECoG power modulation during the DMTS task. Such a map could be used to optimally plan the sequence of ESM mapping. For this analysis we used data from the full trial and full dataset (i.e., data presented in Figure 3) and the beta&gamma model. As in the main analysis we estimated GLM weights on a training dataset and applied those weights to a test dataset. However unlike in the main analysis, here the training dataset comprised all electrodes from nine patients, and the testing dataset comprised all electrodes from the remaining tenth patient. Thus, this analysis could be performed for a new patient using data available before the ESM. After calculating the prediction of all electrodes in the dataset the AUROC for this procedure was 0.73, as compared to 0.76 using the K-fold approach, in line with the results from the standard analysis.

**FIGURE 6:**
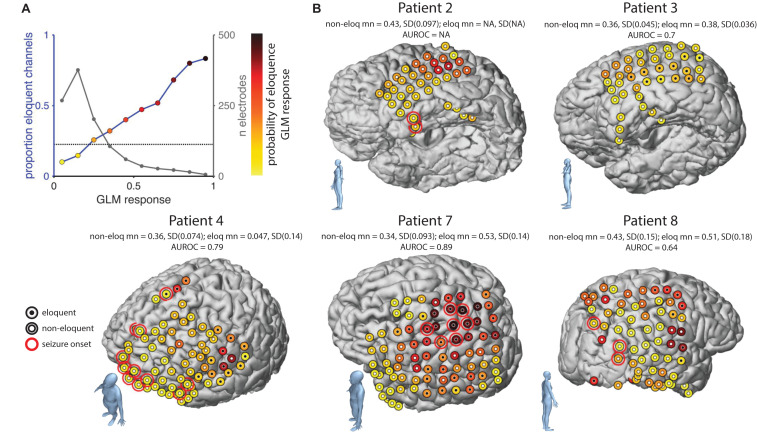
Probabilistic maps of eloquence. **(A)** Relationship between GLM response (i.e., prediction of the binomial probability that an electrode is eloquent) and probability of eloquence (leftward axis) and numbers of electrodes (rightward axis). Dot colors indicate color-scale used in other panels. **(B)** Electrode locations projected onto the individual patient MRI in five patients. Dot color indicates GLM response, dot centers indicate ESM results, white: non-eloquent, black: eloquent. Red rings; seizure onset zones. Bodies in the lower left of each panel indicate the brain orientation. Values above each brain show the quantification of the performance of the GLM. This is given in terms of the mean and standard deviation GLM response of ESM positive (eloquent) and negative (non-eloquent) electrodes, and an AUROC value where patients have both positive and negative electrodes. Non-eloq, non-eloquent; mn, mean; SD, standard deviation; eloq, eloquent; AUROC, area under the receiver operating characteristic curve.

The GLM response corresponds to a prediction of the binomial probability that an electrode is eloquent or not. To visualize the relationship between GLM prediction and the empirical probability of eloquence we binned electrodes into 10 equally spaced bins (with 50% overlap) according to the GLM response. Figure 6A shows the number of electrodes per bin (black line, rightward Y axis) and the proportion of those electrodes with a positive ESM response (blue line and colored dots, leftward *Y*-axis). The proportion of eloquent channels across the whole population is shown by horizontal line. We then showed how the GLM could be used to map five representative patients. The probability of eloquence was represented by color-coding the electrode locations, with darker-red colors indicating higher probability of eloquence (see dot colors in Figure 6A). For comparison, electrodes that were eventually labeled as eloquent by the ESM were marked with a central black dot while electrodes eventually labeled as non-eloquent were marked with a central white dot. For completeness, seizure onset sites were labeled with a red ring. Patient 4 and 8 appear to have a good match between ESM and ECoG prediction, in that darkest colored dots also have black centers. Eloquent areas were far from seizure onset zones, allowing a safe resection. Patient 7 also had a good match between ESM and the ECoG prediction, however, the overlap between eloquent areas and seizure onset precluded a safe resection. Patient 3 showed a good match between ESM and ECoG, however, notice that the estimated probabilities were generally low in this patient such that ESM-positive electrodes were found at a level of ECoG response that would be negative in other patients. This observation indicates that a fixed diagnostic threshold applied to all patients may not be the ideal approach. Patient 2 showed the reverse pattern. In this patient, no eloquent electrodes were found with ESM despite quite high ECoG responses. In this patient the seizure onset zone was located at the temporal pole, while ESM results do not contra-indicate a full resection of the temporal lobe, the ECoG response suggests active cortex above the 5th row of the grid. Taken together, results from the probabilistic map suggest that such a map might be useful as an additional source of information when planning a resection for the removal of epileptogenic tissue, while preserving regions with high ECoG responses.

## Discussion

We found that activity in the alpha, beta and gamma bands could be used to identify eloquent cortex at above chance level, and with rates in line with previous reports (Arya et al., 2018). In line with our hypothesis, combining frequency bands via a generalized linear model (GLM) enhanced the information given by each band alone, thereby increasing the performance in predicting ESM results. Combining beta and gamma was found to be more useful than combining alpha with gamma. Time-resolved analysis showed that the different frequency bands alternated as the “better” measure throughout the trial. Likewise segregating the signal from the eloquent electrodes into cortical *input* and cortical *output* groups showed that the relative performance of alpha, beta and gamma depended on the cortical area, whereby gamma gave better results in *input* areas and beta band gave better results in *output* areas, in line with previous results by Wu et al. (2010). These analyses reveal that the identification of ESM positive electrodes with a specific frequency band (i.e., alpha, beta or gamma) or any frequency combination are not mutually exclusive options. Moreover, our analysis suggests that whether alpha, beta or gamma power are the “better” measure, will depend on several factors, including the patient’s implantation scheme, the task performed, and the selected analysis window. Thus, depending on the implantation scheme, some patients might have additional benefits in the identification of ESM positive electrodes by including beta power modulation. Our GLM approach cuts through this complexity because, regardless of which band provided the better diagnostic ability, the combined approach allowed a performance at least as good as the best individual frequency band and typically offered a significant improvement.

Previous studies have investigated whether ECoG recording could be used as an alternative to ESM by setting a significant response as a diagnostic criterion for identifying eloquent cortex. Here we investigated the diagnostic ability without implementing a diagnostic test. After demonstrating that ECoG responses can reliably predict the probability of a positive ESM response, we showed how a probabilistic map could be constructed that could be used to guide ESM. Guided ESM may reduce the time and effort required of both the patient and clinical team to stimulate all possible neighboring pairs of electrodes. The time-consuming aspect of ESM becomes especially problematic as the number of electrodes increases, for example when using high-density grids. These are becoming more widely used as they increase the spatial resolution of the mapping procedure, thereby increasing surgical precision and decreasing the risk of postsurgical neurological deficits (Escabí et al., 2014). However, cortical mapping using ESM in a high-density grid is time consuming and becomes impractical. In contrast, identifying eloquent cortex using the frequency modulation responses during a cognitive task processes all channels simultaneously. Thus, this approach has the potential to significantly improve the general time efficiency of the mapping procedure. Because ESM can cause seizures, after-discharges (Blume et al., 2004; Aungaroon et al., 2017) and, in some cases, pain (e.g., in the proximity of parieto-opercular cortex; Mazzola et al., 2012), frequency modulation mapping also reduces the chance of stimulation side-effects. Our procedure to create a probabilistic map could be used to guide ESM mapping in a high-density grid such that boundaries between eloquent and non-eloquent cortex could be identified with ECoG and confirmed with ESM.

Although the DMTS task was not designed for cortical mapping, we found that these data were able to be used to identify the eloquent electrodes with performance similar to that reported in previous studies. This aspect points to the wide cortical network that is recruited even when performing a relatively simple task. The used DMTS task involved auditory processing of the syllable, maintenance of the auditory stimulus in short term memory, visual perception of the written cue, comparison of the auditory and visual stimuli for a match-to-sample decision, a motor response, and error monitoring post-response. It hence engages multiple different relevant stages of cognition. Nevertheless, we do not argue that the DMTS task used here is the optimal task. An optimal task would presumably include a wider range of motor actions (our task included only the index and middle finger of the right hand), a wider range of visual stimuli (to activate e.g., face-, place-, and motion-sensitive areas), and a language production section—and has to be feasible in the limited time the patient is available. Our main finding is a proof of concept that the inclusion of low frequency bands, especially the beta band, improves the identification of eloquent electrodes. We would expect that a similar analysis in a dataset acquired during the performance of a structured cognitive task explicitly designed to activate eloquent cortex would result in better performance of ECoG mapping.

The possibility also exists, however, that including low frequency power was only useful in the context of a non-optimal task. It may be that, in an optimal task all eloquent cortices may be sufficiently activated, such that they can be readily identified using the gamma band alone. Our finding that the beta band was more useful in identifying *output* areas than *input* areas argues against this possibility, since the task was arguably better tuned for identifying input areas than *output* areas (e.g., auditory cortex was adequately activated, while IFG (Broca’s area) was only weakly involved during covert rehearsal and reading). Ultimately, additional data or re-analysis of data collected by other groups will be required to clarify this aspect.

In order to better understand the validity of cortical mapping with ECoG, it is relevant to evaluate whether false positives identified by ECoG mapping are in fact false positives or perhaps may be ESM false negatives. The true test of whether an area is eloquent or not is the effect of surgical resection of that area on the neuropsychological function. Currently, ESM is the best predictor of the effect of resection (Arya et al., 2018), however to control for the possibility of false negatives in ESM, it will be important to evaluate the cognitive performance of a large cohort of patients after surgical resection. A large, multi-center effort will likely be required to identify false positive and negative electrodes after both ECoG and ESM mapping. These data were not available in our study.

In our analysis we used the power of gamma, alpha and beta bands as indicators for the eloquence of cortex beneath subdural electrodes. However, with the cross-validation procedure implemented in our analysis it is possible to include a multitude of indicators. Other factors that might further improve mapping could include power, adding modulations from other frequency bands such as theta or delta (Daume et al., 2017; Grooms et al., 2017), power modulations recorded during additional tests, and responses measured in other modalities (e.g., functional mapping using fMRI; Picht et al., 2013; Trimmel et al., 2019, 2020—or transcranial magnetic stimulation). Prior predictions about the likelihood of eloquence at a particular cortical area may also be of interest and help to increase mapping accuracy.

Given the relatively small number of patients included in the present study and the task used, which was developed to study language processing rather than cortical mapping, the method described is a proof of concept not ready to be used as a diagnostic clinical test. In order to further develop our findings into a reliable diagnostic test it will be important to replicate our analysis in a larger patient population, e.g., multi-center clinical trial, in which we would be willing to contribute. It is also important to compare the results with the presence of neurological or cognitive impairments after neurosurgical resection. The optimized ECoG test should clearly outperform ESM in predicting presence of neurological or cognitive impairments after neurosurgical resection. However, inspection of the probabilistic maps seems to argue against this scenario since the maps suggest that between-patient variability in the overall level of ECoG responsiveness is too large to instigate a universal diagnostic criterion. Thus it is more likely that any future ECoG test will be of use in addition to, or as a screening before, ESM.

## Conclusion

Using advanced signal analysis in combination to functional and attentional specific analysis we have shown that including alpha and especially beta power modulations from a DMTS task can improve the diagnostic ability in the identification of eloquent cortical areas over the use of gamma-band power alone. We provided a method to construct probabilistic maps of eloquence based on cortical activity during a DMTS task that can be used as a clinical tool to optimize the planning of electrical cortical stimulation mapping. We conclude that cortical mapping with power modulation is a useful clinical tool to identify potentially eloquent cortical areas (ESM positive) but does not replace the need for electrical cortical stimulation mapping. Further studies using tasks specifically designed for eloquent cortical function identification, the use of tailored individual frequency bands, and comparison of different models with post-resection outcomes will elucidate whether this approach could replace or enhance ESM in clinical settings.

## Data Availability Statement

According to patient’s clinical data privacy conditions, deidentified copies of the raw data supporting the conclusions presented in this study can be provided upon reasonable request. Please contact the corresponding author via email with any inquiries about the data. Custom scripts were written for data reading and visualization and are available upon request.

## Ethics Statement

The studies involving human participants were reviewed and approved by the Medical Ethical Committee of the Maastricht University Medical Center, Maastricht, Netherlands. The patients/participants provided their written informed consent to participate in this study.

## Author Contributions

MEA-M: conceptualization, data curation, resources, software, formal analysis, validation, investigation, visualization, methodology, writing—original draft, and writing—review and editing. GV: software, formal analysis, validation, methodology, writing—original draft, and writing—review and editing. EG: data curation, resources, software, and writing—review and editing. JC: resources, software, and writing—review and editing. SO, JP, and JR: data curation and writing—review and editing. RR: data curation, resources, and writing—review and editing. OS and JD: resources and writing—review and editing. MH, WC, DH, and BJ: writing—review and editing. VK-M: conceptualization, data curation, formal analysis, validation, investigation, resources, writing—original draft, and writing—review and editing. MR: conceptualization, data curation, software, formal analysis, validation, visualization, methodology, writing—original draft, and writing—review and editing. All authors contributed to the article and approved the submitted version.

## Conflict of Interest

The authors declare that the research was conducted in the absence of any commercial or financial relationships that could be construed as a potential conflict of interest.

## Footnotes

1 http://surfer.nmr.mgh.harvard.edu/

2 www.itksnap.org

3 http://www.bioimagesuite.org

## Abbreviations

DMTS: Delayed match-to-sample
ESM: Electrical Stimulation Mapping
ROC: Receiver Operating Characteristic curve
AUROC: Area Under the Receiver Operating Characteristic curve
GLM: Generalized Linear Model.
