# Supplementary material for: Combining Gamma With Alpha and Beta Power Modulation for Enhanced Cortical Mapping in Patients With Focal Epilepsy
Source: Front Hum Neurosci. 2020 Dec 21;14:555054. doi: 10.3389/fnhum.2020.555054 (PMC7779799; doi:10.3389/fnhum.2020.555054)
Supplement: Supplementary file 1 [file Data_Sheet_1.pdf]

Supplementary material for

Combining gamma with alpha and beta power modulation for enhanced cortical mapping in patients with focal epilepsy

Mario E. Archila-Meléndez*, Giancarlo Valente, Erik D. Gommer, João M. Correia, Sanne ten Oever, Judith C. Peters, Joel Reithler, Marc P.H. Hendriks, William Cornejo Ochoa, Olaf E.M.G. Schijns, Jim T.A. Dings, Danny M.W. Hilkman, Rob P.W. Rouhl, Bernadette M. Jansma, Vivianne H.J.M. van Kranen-Mastenbroek, Mark J. Roberts

*Corresponding author. E-mail: mario.archila@tum.de

Published on Front. Hum. Neurosci. | DOI: 10.3389/fnhum.2020.555054

1 Supplementary Materials and Methods

1.1 Electrode implantation procedure

The specific electrode implantation scheme was strictly chosen according to the individual patient protocol depending on the hypothesis of the probable location of the epileptogenic zone, as established by the multidisciplinary epilepsy surgery-working group of the Academic Center for Epileptology. If clinically indicated, language and verbal memory lateralization was established preoperatively using fMRI or a Wada test (Wada and Rasmussen, 2007). The number of implanted electrodes per patient varied, with the number of electrodes' contact points ranging between 24 and 88 per patient. Henceforth we will use the word *electrode* to refer to the electrode's contact point. The day after implantation, a computer tomography and a 3Tesla magnetic resonance imaging scan of the brain were acquired to verify the location of implanted electrodes and to exclude post-surgical complications. These images were used for the reconstruction of the patients' brain surface (native brain space), ~~and~~ the location of the electrodes over the cortical surface and the probabilistic maps of eloquence, see Figure S1 and quantification of the Generalized Linear Model (GLM), See table S1 and section "1.5 Generalized linear model (GLM) fitting and validation" below.

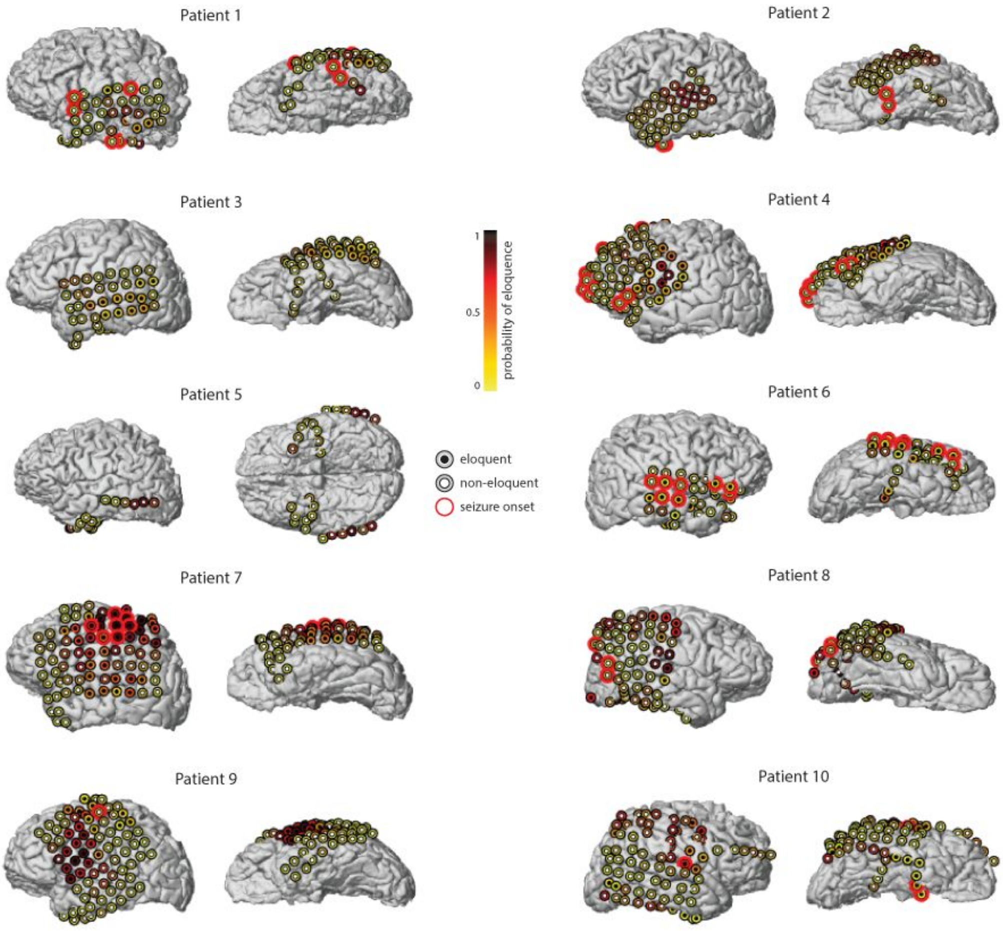

Figure S1 | Probabilistic maps of eloquence for all patients. Electrode implantation schemes projected onto the individual patient (MRI native space) for all patients included in the study. Dot colour indicates the probability of eloquence given by the GLM response, dot centres indicate ESM results, white: non-eloquent, black: eloquent. Red rings; seizure onset zone.

Table S1 | GLM performance and AUROC per patient.

**Patient**	**Non-eloquent**		**Eloquent**		**AUROC**
	**mean**	**sd**	**mean**	**sd**	**value**
1	0.35	0.093	0.41	0.052	0.82
2	0.42	0.103	-	-	-
3	0.36	0.045	0.39	0.036	0.70
4	0.36	0.074	0.47	0.143	0.79
5	0.44	0.155	-	-	-
6	0.35	0.045	0.37	0.059	0.61
7	0.34	0.103	0.54	0.159	0.89
8	0.43	0.153	0.52	0.182	0.64
9	0.32	0.102	0.58	0.232	0.87
10	0.41	0.120	0.40	0.127	0.48

Quantification is given in terms of the mean and standard deviation GLM response of ESM positive and negative electrodes, and an AUROC value where patients have both positive and negative electrodes.

1.2 ECoG data collection

ECoG signals were recorded at 2048 Hz sampling rate using Brain RT software (version 2.0.3164.0, OSG BVBA, Rumst, Belgium). Depending on the total number of electrodes, one or more 64-channel Brainbox EEG-1166 amplifiers (Braintronics B.V., Almere, Netherlands) were used to record from subdural electrodes (Ad-Tech Corporation, Racine, WI, USA) that consisted of platinum alloy discs embedded in a flexible silicon sheet. Electrodes had an exposed surface with a diameter of 2.3 mm. The electrodes were arranged in strips or grids with interelectrode center-to-center distance of 10 mm. As a common reference, an inactive scalp electrode located over the forehead was used at the start of the recording and was in most cases changed to a relatively silent (i.e., not showing any epileptic activity during seizures) implanted electrode after the first seizure was recorded.

1.3 Electrical-cortical stimulation mapping (ESM)

In the current study an electrode was considered eloquent if it generated symptoms during bipolar electrical stimulation (i.e., electrical stimulation across two electrodes) in at least two neighbouring electrode-pairs in any direction (i.e., horizontal, vertical or diagonal).

The ESM proceeded as follows: the stimulation of an electrode-grid was planned by selecting non-continuous electrode pairs (Figure S2). Bipolar electrical stimulation was initially applied between a planned pair of electrodes (e.g. electrode pair 1-2 in Figure S2.A). If no neurological symptoms or function disturbances were observed, electrical stimulation was applied to the next planned pair (e.g., pair 3-4), which included neither of the electrodes included in the first pair. If instead, symptoms were generated by the electrical stimulation of the first pair (e.g., pair 1-2) then, electrical stimulation was applied to a neighbouring pair of electrodes that included one of the previously stimulated electrodes and (if possible) one functionally silent (i.e., not generating symptoms) electrode (e.g. pair 1-5). If this pair (e.g. pair 1-5) resulted in symptoms (Figure S2.B), then electrode 1 would be labelled as eloquent. If a pair that includes the second electrode of the first pair (e.g. pair 2-3) resulted in no symptoms, that electrode would be labelled as non-eloquent. In a different scenario (Figure S2.C), if pair 1-5 results were negative and pair 2-3 results were positive, electrode 1 would be labelled non-eloquent and electrode 2 would be labelled eloquent. In some cases, it is possible that the stimulation of an electrode-pair located at the border of the grid (e.g., pair 1-2) results in reproducible symptoms, but the stimulation of all neighbouring pairs (i.e., pairs 1-5, 2-3, and 2-6) do not generate any symptoms. In such cases at our center, for clinical decision making we assume both electrodes (i.e., 1 and 2) to be eloquent. This approach minimizes the risk of resecting eloquent cortex.

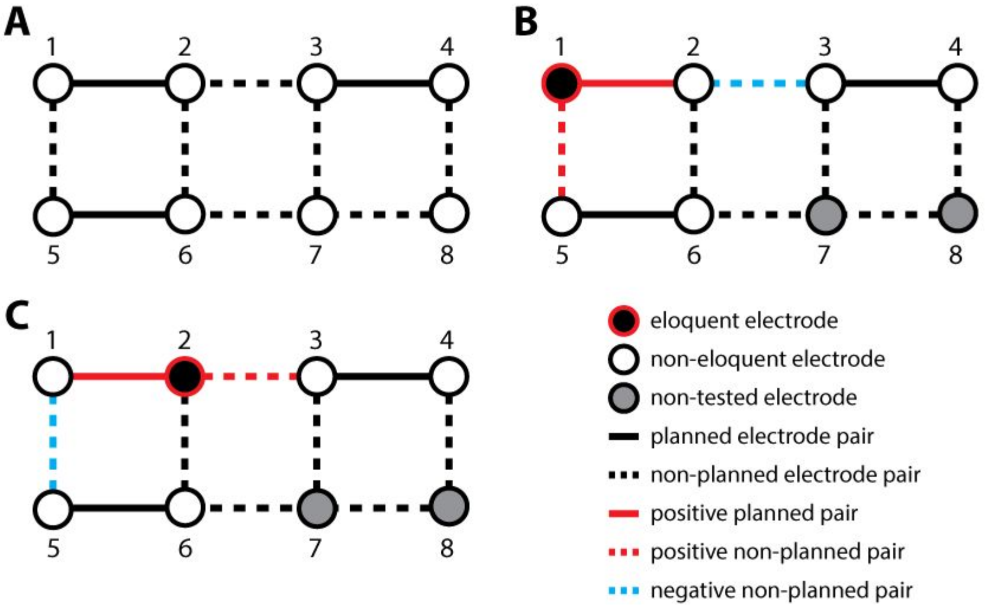

Figure S2 | Schematic representation of two possible ESM cases in a toy example of a 2 by 4 electrodes ECoG grid. **(A)** Planned stimulation pairs. **(B)** Case one, in which electrode 1 is found to be eloquent after symptoms are produced during electrical stimulation at pair 1-2 and 1-5, but not 2-3. **(C)** Case two in which electrode 2 is found to be eloquent after symptoms are produced at pair 1-2 and 2-3 but not 1-5.

The stimulation was performed with a constant current stimulator (Osiris cortical stimulator, Inomed, Emmendingen, Germany) with the following stimulation settings: trains of square wave pulses, pulse frequency 50Hz, pulse duration 0.2 milliseconds, with a train duration of 3 to 7 seconds (depending on the function tested and the specific patient conditions). The current was increased in (1-)2 mA steps to a maximum of 15 mA (e.g., 1 mA, 3 mA etc.). During ESM for language testing, participants performed a reading task and, if reading was impaired by the electrical stimulation procedure (e.g., speech arrest (anarthria), hesitation, distortion), testing was extended with additional tasks (e.g., naming, counting, tongue movement task). ESM was stopped if any of the following end-points was reached: 1) a clear and reproducible generation of neurological symptoms, 2) impairment of any of the performed (cognitive) tasks, or 3) reaching the maximum stimulus intensity of 15 mA without causing symptoms, deficits or after-discharges. This procedure was then repeated at the next planned electrode pair. Stimulated electrodes that resulted in neurological signs or symptoms or cognitive impairment were labelled as *eloquent* and assigned to one of nine functional categories defined by the clinical neurophysiologists (i.e., motor, sensory, mixed sensorimotor, language temporobasal, language Broca, language Wernicke, emotion, visual, and auditory). This categorization was used as the ground truth against which our identification results were compared during the subsequent analyses.

All tests were performed in the epilepsy-monitoring unit (EMU) by an expert clinical neurophysiologist (V.H.K.M. or D.M.W.H). The selection of electrode pairs for stimulation and the total number of electrodes stimulated per session was decided individually for each participant by the clinical neurophysiologist. For the remainder of the text we use the term ‘eloquent’ interchangeably to refer to areas of cortex and

to ESM positive electrodes, under the assumption that those electrodes correspond to eloquent cortex.

1.4 Delayed match-to-sample task

A trial consisted of a speech sound stimulus comprising a spoken consonant-vowel syllable of 340 milliseconds duration followed, after a jittered interval of 550-750 milliseconds, by a written cue displayed for 500 milliseconds. After the presentation of the written cue, a 1500 milliseconds period was allowed for a button response followed by an inter-trial interval (ITI) of 1000 milliseconds as baseline before the next trial began (See Figure S3, task structure). The written cue was either a complete syllable, a vowel, or consonant letter, depending on the specific block. The cue either matched or did not match the previous sound stimulus. Participants were asked to compare the syllable sound with the written cue and respond 'match' or 'mismatch' as quickly and accurately as they could. The stimuli matched in 50% of the trials, and 'match'/'non-match' trials were balanced across conditions and randomized per participant. The identical sound stimuli were also presented in a passive listening condition in which sound onsets were jittered by 900-1100 ms while participants held their gaze steady on the computer screen. Stimuli were presented in the epilepsy-monitoring unit using a laptop computer with built-in open-field speakers. Stimulus and behavioural event triggers were sent to the clinical data recording equipment via a parallel port to the Ethernet interface box (Ethernet-102 V2, Braintronics B.V., Almere, Netherlands). All behavioural responses, stimuli, event identities, and timings were presented and logged using Presentation (Neurobehavioral Systems; www.neurobs.com, RRID: SCR_002521). The task was performed in 2 to 6 sessions per patient, in which trials were grouped into 4 blocks of 54 trials, with each block representing a different attention condition. Sessions lasted approximately 15 minutes and 1 to 2 sessions were performed per day. The total experiment time across sessions was between 30 and 90 min per patient.

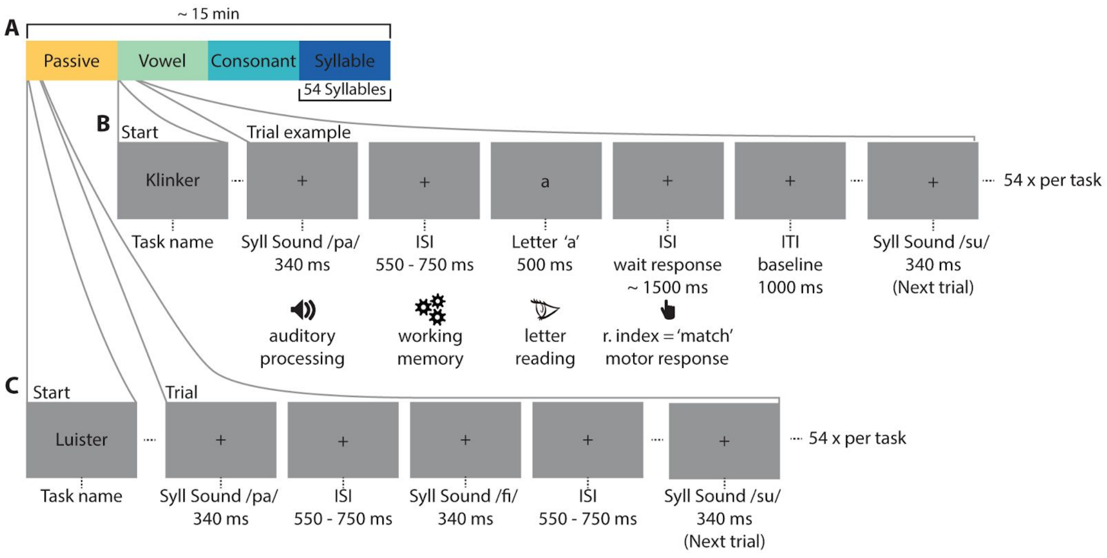

Figure S3 | Task structure. **(A)** Schematic representation of one session comprising four tasks. Each task block consisted of 54 trials. The duration of the tasks is schematically represented by the length of the four coloured blocks. Each task began with an instruction display in dutch (i.e., 'Vowel' for the attend to vowel, 'Consonant' for attend to consonant, 'Syllable' for attend to syllable, or 'Listen' for the passive listening task). **(B)** Example of an

individual match trial in an active task (i.e., attend to vowel). **(C)** Example of an individual trial in the passive listening task.

1.5 Generalized linear model (GLM) fitting and validation

In order to combine the contribution of different bands in a relatively straightforward and interpretable manner, we framed the identification problem as a linear regression, where the independent variables are the different bands and the dependent variable is the ESM response. The ESM response can be either 0 or 1, therefore a standard linear regression would be unsuited since the assumption of normality of the residuals is violated. We therefore resorted to a Generalized Linear Model (GLM), a generalization of linear regression models to cases where the error distribution is not normal (Nelder and Wedderburn, 1972; McCullagh and Nelder, 1998). We used a binomial likelihood and logit link function (McCullagh and Nelder, 1998), where for each electrode the dependent variable (y) was the ESM response (binary, either eloquent or non-eloquent) and the predictors were the ECoG power change in each frequency band. Once the model was estimated, we could determine the optimal weighting of the power change at each frequency band as regression coefficients to predict the ESM response. Since calculations of AUC using the same data used for GLM estimation would result in an inflation of type-I errors, we estimated the GLM coefficients on a subset of electrodes and determined the area under the receiver operating characteristic curve on the remaining portion. Thus, we first partitioned the eloquent and non-eloquent electrode categories into k disjoint datasets and used the data contained in k-1 partitions (i.e., leaving one partition out for testing later the model's prediction) to estimate a GLM having ECoG power change in each frequency band as independent variables x, and eloquent or non-eloquent ESM categories as the dependent variable y (y being 0 for non-eloquent electrodes, and 1 for eloquent electrodes). Both quadratic and interaction terms were included in the estimation in order to benefit from possible non-linearities in the ECoG response (i.e., power change) and for possible interactions among the two. The model was then used to predict the ESM response in the left-out partition. The whole procedure was repeated for all the partitions (i.e., k-fold cross-validation, in our case we used $k = 10$). The resulting prediction, concatenated across all the test partitions, was compared with the ground truth ESM by means of area under the receiver operating characteristic (ROC) curve. Finally, the whole procedure was repeated 20 times, randomly selecting the partitions, and the results were averaged. When considering each frequency band alone, we performed a similar analysis including the quadratic terms, but using only the one predictor of interest.

1.6 Area under the receiver operating characteristic curve analysis (AUROC)

It is important to note that we did not implement a diagnostic test, for which we believe more data, and an optimized behavioural task, would be required. Previous studies have quantified the sensitivity (i.e., true positive divided by the sum of true positive and false negative) and specificity (i.e., true negative divided by the sum of true negative and false positive) of changes in cortical power. However these values are determined by both the diagnostic ability and the applied discrimination threshold.

The diagnostic ability may be considered significant if the AUROC is different from chance. In our analysis, AUROC was higher than 0.5 when the response (i.e., power change within a given frequency band) in eloquent electrodes was greater than in

non-eloquent electrodes, and less than 0.5 when the response in eloquent electrodes was lower than the response in non-eloquent electrodes. A value of 0.5 indicated no difference between eloquent and non-eloquent channels. Significance was therefore tested using a two-tailed test, where the null distribution was empirically calculated by randomly permuting (1000 times) the labels between eloquent and non-eloquent electrodes and calculating the corresponding AUROC from the permuted data. AUROC values greater than the 97.5% quantile of the distribution or less than the 2.5% quantile of the distribution were considered significant.

1.7 Bootstrap procedure for statistical testing receiver operating characteristic between models

Statistical comparisons were then carried out by comparing the differences between models. Here we made two types of comparison. First, we tested the performance of each model compared with chance performance (0.5) by means of bootstrapping. We resampled (with replacement) the channels and considered, for each resampled dataset, the obtained AUROC value. We deemed a model significant when 95% of the bootstrapped AUROC values were larger than 0.5 (i.e. when chance was outside the left one-sided 5% confidence interval). Next, we tested the difference in performance between models, which was done again by implementing a bootstrapping procedure across electrodes. To determine whether a (combined) model resulted in an improvement over the gamma-only model, we made a pairwise comparison by calculating the difference in performance between that model and the performance of the gamma only model, across all the randomly drawn sets of electrodes. An improvement over the gamma-only model was considered significant if >95% of the bootstrapped datasets showed a positive difference.

Supplementary References

McCullagh, P., and Nelder, J. A. (1998). *Generalized linear models*. 2nd ed. Boca Raton: Chapman & Hall/CRC.

Nelder, J. A., and Wedderburn, R. W. M. (1972). Generalized Linear Models. *Journal of the Royal Statistical Society. Series A (General)* 135, 370. doi:10.2307/2344614.

Wada, J., and Rasmussen, T. (2007). Intracarotid injection of sodium amytal for the lateralization of cerebral speech dominance. 1960. *J. Neurosurg.* 106, 1117–1133. doi:10.3171/jns.2007.106.6.1117.
